# Long-Term Outcomes After Kidney Transplantation in a Recipient With Birt–Hogg–Dubé Syndrome

**DOI:** 10.1155/crit/5889953

**Published:** 2025-07-23

**Authors:** Armando Salim Munoz Abraham, Adriana Medina, Rojin Esmail, Timothy Trestrail, Franco Cabeza Rivera

**Affiliations:** ^1^Department of Surgery, University of Miami Miller School of Medicine, Miami Transplant Institute, Jackson Memorial Hospital, Miami, Florida, USA; ^2^University of Miami Miller School of Medicine, Jackson Memorial Hospital, Miami, Florida, USA; ^3^Department of Surgery, University of Miami Miller School of Medicine, Jackson Memorial Hospital, Miami, Florida, USA; ^4^Department of Medicine, Division of Nephrology, Miami Transplant Institute, University of Miami Miller School of Medicine, Jackson Memorial Hospital, Miami, Florida, USA

**Keywords:** Birt–Hogg–Dubé syndrome, cancer, dialysis and transplantation, genetics, renal cell carcinoma

## Abstract

**Introduction:** Birt–Hogg–Dubé syndrome is a rare autosomal dominant disorder caused by folliculin germline mutations. Renal cell carcinoma is the most serious manifestation of this condition occurring at a rate of 30%, often requiring nephrectomy. Although preserving renal function remains the central goal of management, the risk of end-stage renal disease remains high. Patients with other inherited renal carcinomas have been successfully transplanted in the past, but there is scarce literature regarding Birt–Hogg–Dubé syndrome and kidney transplantation.

**Case Presentation:** A 48-year-old male presented to our facility for evaluation of recurrent pneumothorax. Computed tomography of the chest revealed bilateral pulmonary cysts and multiple bilateral renal masses. Given the coexisting pulmonary cysts and renal masses, he was diagnosed with Birt–Hogg–Dubé syndrome. Bilateral radical nephrectomy was performed due to the presence of multifocal tumors measuring up to 5 cm. Tumor pathology was consistent with oncocytoma and renal cell carcinoma. After 2 years of hemodialysis and surveillance, the patient underwent kidney transplant. At 2-year follow-up after transplantation, renal function remains stable and has no evidence of recurrent renal disease, managed with belatacept and mTor inhibitors.

**Discussion:** Tumor aggressiveness, metastasis risk, and time in remission are important factors when evaluating a patient with a history of Birt–Hogg–Dubé syndrome associated with renal cell carcinoma for kidney transplant. Therefore, these patients are suitable candidates for transplant after a minimum waiting period. Posttransplant immunosuppression with mTOR inhibitors can be considered since the mutation of the tumor suppressor folliculin germline in the mTOR pathway is central to Birt–Hogg–Dubé syndrome pathogenesis.

**Conclusion:** In this case report, we demonstrated that kidney transplantation is a viable option for patients with Birt–Hogg–Dubé syndrome–related renal cell carcinoma.

## 1. Introduction

Birt–Hogg–Dubé syndrome (BHDS) is a rare autosomal dominant disorder resulting from mutations in the folliculin (FLCN) gene. This syndrome can manifest as fibrofolliculomas, development of lung cysts, recurrent pneumothorax, and renal malignancies. Renal cell carcinoma (RCC) is one of the most serious manifestations of BHDS, which occurs at a rate of 30% [1].

Despite renal preservation efforts, many of these patients develop advanced kidney disease or end-stage renal disease (ESRD) because of unilateral or bilateral nephrectomies required for tumor excision. While kidney transplantation remains the treatment of choice for patients with ESRD, there is an increased risk of developing malignancies attributable to the immunosuppressive therapy.

Successful transplantation has been recorded in patients with genetically inherited RCCs (e.g. von Hippel Lindau disease), but literature on renal transplantation in patients with BHDS-associated RCC is notably scarce [2]. In this report, we present our experience with a BHDS patient diagnosed with bilateral RCC who underwent a renal transplant 2 years postbilateral nephrectomy and remains disease-free.

## 2. Case Presentation

The patient is a 48-year-old male with a history of hypertension who presented to our facility for evaluation of recurrent pneumothorax in 2020. A chest computed tomography scan revealed multiple bilateral pulmonary cysts and incidentally detected multiple bilateral renal masses. These masses were later confirmed as radiographically consistent with RCC by a subsequent magnetic resonance imaging (MRI). The combination of numerous bilateral pulmonary cysts, recurrent pneumothorax, and bilateral renal masses suggested a variant of BHDS without cutaneous manifestations. Genetic testing confirmed a mutation in the FLCN gene, CASR and RAD51C (variant of uncertain significance). Interestingly, despite the confirmed diagnosis, the patient's family history was negative for renal malignancy or any previous diagnosis of BHDS.

**Figure 1:**
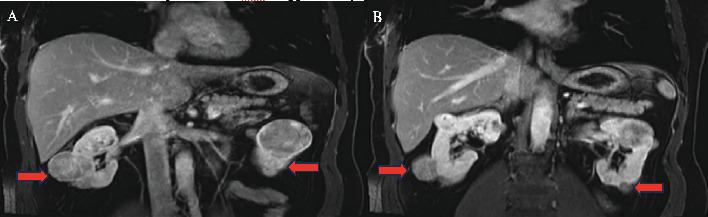
(A, B) CT scan showing bilateral renal tumors.

Following a comprehensive evaluation and informed discussion, the patient underwent bilateral radical nephrectomy due to the presence of multifocal tumors measuring up to 5 cm and subsequently began hemodialysis. The pathological examination of the excised tumors identified the presence of bilateral renal oncocytomas and chromophobe RCC. Specifically, the pathology report describes for the left kidney, multiple renal tumors (more than 10), composed of a mixture of chromophobe RCCs and oncocytomas, and for the right kidney, multiple renal tumors (does not specify how many) composed of a mixture of chromophobe RCC and oncocytoma. The disease was staged as pT1bN0M0 (Figure 1).

Then, 2 years following the bilateral nephrectomy, the patient displayed no signs of malignancy. A PET scan was performed 2 years after the bilateral nephrectomies and did not show any evidence of residual or recurrent disease. The patient was deemed a suitable candidate for kidney transplantation, and after clearance by medical oncology, he underwent a deceased donor kidney transplant (DDKT).

Despite initial slow graft function, the patient experienced an overall uneventful recovery. Our induction protocol therapy at the time of the surgery consisted of thymoglobulin 1 mg/kg for two doses with the addition of basiliximab 20 mg and methylprednisolone. Our maintenance immunosuppressive therapy consisted of a steroid-free regimen that included tacrolimus and mycophenolate starting on postoperative day one. Eventually, the patient was transitioned to a belatacept- and everolimus-based regimens to minimize the future risk for malignancies. Serial creatinine measurement follow-up has demonstrated adequate kidney function, with stable levels of 1.9 mg/dL at 6 months, 1.7 mg/dL at 1 year, and 1.7 mg/dL at 2 years. His immunosuppression regimen remains with belatacept and everolimus, due to the prior RCC. No pneumothorax or evidence of recurrent malignancy has been reported since the kidney transplantation.

## 3. Discussion

BHDS, an infrequent autosomal dominant condition, carries an association with FLCN mutations. The FLCN gene is located on the short arm of chromosome 17 [3]. The function of FLCN is hypothesized to form a complex with the proteins FNIP1 and FNIP2. This complex is thought to interact with the 5⁣′ AMP-activated protein kinase, thereby acting as a negative regulator of the mTOR pathway and functioning as a tumor suppressor. The FLCN gene is expressed in nearly all cell types, with notable presence in skin cells, distal nephrons, and Type 1 pneumocytes [3–5].

This syndrome exhibits a heterogeneous phenotypic presentation, even among family members carrying the same FLCN mutation [4]. Characteristics commonly associated with BHDS, found in 85% and 70%–80% of affected individuals, respectively, include fibrofolliculomas and bilateral pulmonary cysts. Additionally, renal tumors are seven times more likely in patients with BHDS than in the general population, with a median diagnosis age of 48 [4, 5]. The renal tumors in BHDS patients are typically small, multifocal, and bilateral, with diverse tumor histologies including chromophobe, hybrid chromophobe/oncocytoma, clear cell carcinoma, and papillary RCC [6]. Prominent among these are chromophobe RCC and hybrid chromophobe/oncocytoma, considered less aggressive with lower metastatic potential compared to clear cell renal carcinoma [7]. Chromophobe and hybrid chromophobe/oncocytoma demonstrate a more indolent metastatic course compared to sporadic renal carcinomas [7, 8].

For patients with BHDS, the treatment approach can vary depending on tumor size. Radio ablation and cryoablation are typically performed for tumors smaller than 3 cm or alternatively, tumor growth rate may be monitored until tumors reach the 3-cm threshold requiring surgical intervention [9–11]. However, due to the technically challenging nature of partial nephrectomies, they may come with a higher risk of postoperative complications, including bleeding, recurrence, and urinary fistulas [12]. For cases involving multiple and larger tumors, in institutions without expertise in partial nephrectomies, radical nephrectomies may be favored.

Because of unilateral or bilateral nephrectomies required for tumor excision, many patients develop advanced kidney disease or ESRD. Although kidney transplantation remains the treatment of choice for patients with ESRD, it comes with a higher risk of cancer than in the general population [13]. Key considerations for transplantation in BHDS patients with ESRD who have previously undergone nephrectomy include tumor aggressiveness, metastasis risk, recurrence rate, and duration of remission post-nephrectomy. Transplant guidelines, including those from KHA-CARI, emphasize the significance of TNM staging and tumor histology in assessing the risk of cancer recurrence and determining transplant eligibility and the cancer-free waiting interval before transplantation [9]. Given chromophobe and hybrid chromophobe/oncocytoma RCCs, which typically exhibit a lower metastatic rate and indolent course, these patients are excellent candidates for renal transplantation when nephron-sparing measures are infeasible due to the number and size of the tumors [7, 8].

The duration of remission before transplantation is crucial, with most guidelines recommending a minimum waiting period after successful cancer therapy for larger, symptomatic tumors [9]. In the present case, the patient had multifocal and bilateral RCC, with pathology revealing a mix of oncocytoma and chromophobe RCC, pT1bN0M0. He remained in remission for 2 years between the bilateral nephrectomy and kidney transplant.

Immunosuppression selection is of paramount importance for any patient undergoing a kidney transplant. In BHDS patients, maintenance therapy with mTOR inhibitors might be considered after the initial wound-healing period but should be done so with caution due to the risk of podocyte apoptosis and potential development of focal segmental glomerulosclerosis. Given that the FLCN mutation, a tumor suppressor of the mTOR pathway, is central to the pathogenesis of BHDS, inhibiting this pathway could potentially decrease the risk of tumor recurrence after transplantation [14].

To date, the only previous references in literature include a case report of one BHDS patient listed for transplant, with the management course remaining unknown, and a second who underwent living donor kidney transplantation before the diagnosis was determined [2, 15].

## 4. Conclusion

BHDS frequently presents with RCC [16]. Management of these cases hinges on factors such as tumor size, number, and overall patient health. In instances where nephron-sparing methods are unfeasible and postsurgical remission is successful, renal transplantation can be a viable option, as demonstrated in our patient's case. Additionally, we highlight the potential benefit of incorporating mTOR inhibitors in the posttransplantation regimen due to the role of the FLCN mutation in the mTOR pathway. While this case contributes significantly to the limited existing literature, more research is necessary to validate these findings and optimize treatment strategies for BHDS-associated RCC.

## Data Availability

The data that support the findings of this study are available from the corresponding author upon reasonable request.
